# The Expression of Hippocampal NRG1/ErbB4 Correlates With Neuronal Apoptosis, but Not With Glial Activation During Chronic Cerebral Hypoperfusion

**DOI:** 10.3389/fnagi.2018.00149

**Published:** 2018-05-23

**Authors:** Yue Hei, Rong Chen, Xicai Yi, Lizhou Wei, Qianfa Long, Weiping Liu

**Affiliations:** ^1^Department of Neurosurgery, Xijing Hospital, Fourth Military Medical University, Xi’an, China; ^2^Department of Neurology, Xijing Hospital, Fourth Military Medical University, Xi’an, China; ^3^Department of Neurosurgery, Institute of Mini-invasive Neurosurgery and Translational Medicine, Xi’an Central Hospital, Xi’an, China

**Keywords:** chronic cerebral hypoperfusion, neuronal apoptosis, glial activation, NRG1, ErbB4

## Abstract

Permanent bilateral common carotid occlusion (2VO) is well-established to investigate the chronic cerebral hypoperfusion (CCH)-induced cognitive deficits. Besides, previous studies suggested that disturbance of Neuregulin1 (NRG1)/ErbB4 signaling is associated with cognitive impairments, as well as neuronal apoptosis and neuroinflammation in CNS. However, the expression pattern of hippocampal NRG1/ErbB4 has not been systematically investigated during CCH. Here, we aim to investigate the temporal changes of hippocampal NRG1/ErbB4 during CCH and their possible relationship with neuronal apoptosis and glial activation. Morris water maze (MWM) and Radial arm water maze (RAWM) tests were used to analyze cognitive impairment in 2VO rats at 28 days post-surgery, and Enzyme-Linked Immunosorbent Assay (ELISA), western blotting and immunostaining were performed at different time points (24 h, 7 days, 14 days, 28 days) to detect the expression pattern of NRG1/ErbB4 and the distribution of ErbB4. Neuronal nuclei (NeuN), NeuN/TUNEL, Iba1 and GFAP immunostaining and caspase activity in hippocampal CA1 subarea were assessed during CCH as well. We found that the expression of NRG1 and phosphorylated ErbB4 (pErbB4)/ErbB4 changed in a time-dependent manner (up-regulated in the acute phase and then decreased in the chronic phase of CCH). Besides, ErbB4-expressed neurons and selective types of GABAergic cells decreased after CCH, but the distribution pattern of ErbB4 remained unchanged. In addition, the expression of hippocampal NRG1/ErbB4 positively correlated with the level of neuronal apoptosis (both NeuN/TUNEL immunostaining and caspase-3 activity), but not with glial activation according to Pearson’s correlation. These findings indicated that hippocampal NRG1/ErbB4 may be involved in the pathogenesis of CCH, especially neuronal apoptosis during CCH.

## Introduction

Chronic cerebral hypoperfusion (CCH) may cause dramatic cognitive decline and neurodegenerative changes that are closely associated with Alzheimer’s disease and vascular dementia (Farkas et al., 2007; Zhao and Gong, 2015), but the underlying mechanisms are poorly understood so far. It is known that hippocampal neuronal death and glial activation contribute to cognitive decline (Farkas et al., 2007; Du et al., 2017) in the permanent bilateral common carotid artery occlusion (2VO) rats (a well-established animal model for CCH). More specifically, neuronal apoptosis and glial activation were found to be directly correlated with CCH-induced cognitive deficits (Xi et al., 2014). Bennett et al. (1998) previously reported selectively increased level of neuronal apoptosis in CA1 subarea during CCH, and a number of studies have further demonstrated that neuroprotection in CA1 region could improve cognitive ability in 2VO rats (Anastacio et al., 2014; Kwon et al., 2015; Liu et al., 2015). Besides, activated microglia and astrocytes take an essential role in neuroinflammation by producing inflammatory chemokines and cytokines (such as IL-1β, IL-6 and TNF-α) (Cechetti et al., 2012; Yang et al., 2014; Du et al., 2017), which may further accelerate the disease progression. These findings suggest the key role of both hippocampal neuronal apoptosis and glial activation in CCH.

Neuregulin1 (NRG1) is a growth factor that contains the epidermal growth factor (EGF)-like domain (Wu et al., 2015). Mature NRG1 is largely diffusible and exerts its effects via tyrosine kinase receptors like ErbB4, which can bind NRG1 and activate downstream signaling (Mei and Xiong, 2008; Guan et al., 2015). NRG1/ErbB4 signaling has been heavily implicated in neuronal migration, synaptic plasticity (Mei and Xiong, 2008), etc. Besides, recent studies have suggested NRG1/ErbB4 signaling may be also involved in cognitive processes, neuronal protection and anti-inflammatory actions (Li et al., 2014; Lu et al., 2016; Tian et al., 2016). With regard to cognitive processes, endogenous hippocampal ErbB4 receptors in Parvalbumin (PV) interneurons were found to mediate NRG1’s action in long-term potentiation (LTP), which is essential for cognitive process (Chen et al., 2010). Furthermore, a number of studies have suggested that NRG1 administration can ameliorate cognitive impairments in cerebral ischemia and neurodegenerative diseases (Rong et al., 2015; Ryu et al., 2016). On the other hand, emerging evidence has demonstrated that NRG1 can effectively protect neurons directly (attenuate neuronal apoptosis) and indirectly (preserve blood-brain barrier integrity and reduce glial activation) (Simmons et al., 2016; Gao et al., 2017). However, all these beneficial effects of NRG1 could be abolished by ErbB4 inhibition (Tan et al., 2011; Guan et al., 2015; Zhang et al., 2017), indicating the role of ErbB4 in NRG1’s actions. Taken together, the expression of hippocampal NRG1/ErbB4 signaling may be associated with cognitive ability, neuroprotection and glial activation in CCH. But the expression of hippocampal NRG1/ErbB4 in CCH has not been systematically investigated so far, as well as their possible relationship with neuropathological changes during CCH.

Therefore, the present study aimed to investigate the temporal changes of NRG1/ErbB4 expression in hippocampus (especially CA1 region) and their possible relationship with neuronal apoptosis and glial activation in a rat model of CCH.

## Materials and Methods

### Animals and 2VO Model

All male Sprague-Dawley rats (230–250 g) were purchased from the Experimental Animal Center of the Fourth Military Medical University. Rats were housed in groups under controlled conditions (12-h light/dark cycle; 22–24°C) with free access to food and water. All experiments were approved by the Ethics Committee of the Fourth Military Medical University and followed the guidelines for the Care and Use of Laboratory Animals of the National Institute of Health (Publication No. 85–23, revised 1996). 2VO was used here. Food and water were withheld for 1 day prior to surgery. Rats were anesthetized with 10% chloral hydrate (300 mg/kg, i.p.). The bilateral common carotid arteries and vagal nerves were gently exposed and separated. Each artery was permanently ligated with silk suture. Then the wound caused by midline ventral incision was carefully sutured and closed. A total of 140 rats underwent the 2VO surgery and 44 of them died within the first 3 days. Rats that underwent a sham operation were treated similarly, but the common carotid arteries were not ligated.

### Behavioral Assessment

Rats (sham group: *n* = 6; 2VO group: *n* = 6) underwent Morris water maze (MWM) tests at 28 days post-operation. The procedure has been described previously (Long et al., 2015). Briefly, the black circular tank was divided into four quadrants (I, II, III and IV) and the platform (1 cm below the water surface) was located in the center of quadrant IV for the hidden platform tasks, during which the rats were released from a randomized quadrant to search for the platform within 60 s. Rats would be gently guided to the platform and took a rest for 10 s if they failed to find it. The escape latency and swimming speed were collected during the five training days for hidden platform tasks. Then we removed the platform and performed the 1-day probe trial. The animals were allowed to swim freely for 60 s in the pool, during which the percentage of time spent in the target and the number of platform crossings were recorded. Apart from this, we also conducted radial arm water maze (RAWM) as described previously (Murray et al., 2011; Penley et al., 2013). This RAWM test is specialized to evaluate the hippocampal-dependent spatial memory ability, and it is a modified version of the traditional land-based tests. There are eight equally sized arms (numbered I to VIII) radiated from a central area (50 cm in diameter) and extra-maze cues are set around the room. As previously described (Murray et al., 2011), the escape platforms were placed at the ends of four arms (I, III, V, VII), and animals were released from the starting arm (II) to find the hidden platform within 120 s. The cumulative data of working and reference memory errors over the 5 days were collected (four trials per day).

### Enzyme-Linked Immunosorbent Assay (ELISA)

Since mature NRG1 is largely diffusible (Mei and Xiong, 2008), the concentration of NRG1 in the hippocampus was evaluated by ELISA at different time points (24 days, 7 days, 14 days, 28 days) during CCH as described in previous studies (Li et al., 2014; Gao et al., 2017). Briefly, the tissue of bilateral hippocampal CA1 from rats (sham group: *n* = 6; 2VO group: *n* = 6 per time point) was carefully isolated under microscope. Afterwards, the tissue was homogenized (using ice-cold water) and centrifuged (12,000 rpm for 5 min) to get the supernatant. The concentration of NRG1 was analyzed using ELISA kit following the manufacturer’s instructions (R&D Systems, Minneapolis, MN, USA). We also tested the concentration of EGF using the specific Human EGF ELISA kit (Abcam, Burlingame, CA, USA) at the same time during CCH, which was described previously (David et al., 2014).

### Western Blotting

In order to detect the dynamic changes of ErbB4 expression in the hippocampal subareas during CCH, rats (sham group: *n* = 6; 2VO group: *n* = 6 per time point) were sacrificed for western blotting analysis at different time points (24 days, 7 days, 14 days, 28 days). The proteins were extracted from the tissue using the BioRad protein assay kit (Hercules, Wilmington, DE, USA). The normalized protein samples were subjected to sodium dodecyl sulfate-polyacrylamide gel, and then transferred to nitrocellulose membranes (Whatman, Germany). The membranes were blocked using 5% skim milk in TBST at room temperature for 1.5 h and incubated with anti-β-actin (Cell Signaling Technology, Danvers, MA, USA), anti-phosphorylated ErbB4 (pErbB4) (Abcam, Burlingame, CA, USA) and anti-ErbB4, anti- phosphorylated ErbB1 (pErbB1) and anti-ErbB1 (Cell Signaling Technology, Danvers, MA, USA) antibodies overnight at 4°C. After incubation at room temperature for 1 h, the membranes were thoroughly washed and incubated with peroxidase-conjugated secondary antibody (Santa Cruz Biotechnology, Santa Cruz, CA, USA) for 1 h. Finally, the labeled proteins were visualized with chemiluminescence (SuperSignal West Pico, Pierce), and the quantification of pErbB4, ErbB4 expression on X-ray films was analyzed using Quantity One 4.5.2 software. The value for the sham group was defined as 100%.

### Immunostaining and Cell Counting

Rats (sham group: *n* = 6; 2VO group: *n* = 6 per time point) were anesthetized with 300 mg/kg chloral hydrate intraperitoneally and perfused using 4% paraformaldehyde. The rat brains were then removed, and routinely dehydrated, embedded in paraffin and cut into 2 μm sections. The sections were incubated overnight with rabbit-anti ErbB4 (1:100, Santa Cruz Biotechnology, Santa Cruz, CA, USA) or mouse anti-ErbB4 (1:50, Abcam, CA, USA), and mouse antibodies of neuronal nuclei (NeuN; 1:200), GFAP (1:200), GABA (1:50), GAD67 (1:100), PV(1:50), Somatostatin (SOM) (1:50; Abcam, CA, USA), or rabbit anti-Iba1 (1:100, Abcam, CA, USA), and then detected with secondary antibodies (goat anti-rabbit Alexa Fluor 488-conjugated and goat anti-mouse Alexa Fluor 594-conjugated) (Invitrogen, Carlsbad, CA, USA) (room temperature for 3 h followed by 0.0001% DAPI (Beyotime, China) staining) to see the distribution and location of ErbB4 receptors. The co-expression of ErbB4 and other markers in the hippocampus (including stratum oriens (SO), pyramidale (SP) and radiatum (SR) according to Paxinos and Watson (2005) was determined by counting the cells that clearly represented with DAPI staining. The images were captured using a confocal microscope (Olympus, Japan; magnification, 200×). Data obtained from at least 10 sections for each rat by three investigators were averaged to get a single estimate for each animal.

### TUNEL Assay

To further determine the co-localization of apoptotic cells and neurons in the hippocampus, immunofluorescent double staining of TUNEL/NeuN was performed. Briefly, the brain sections were prepared as above. Sections were incubated in permeabilization solution (0.1% Triton X-100, 0.1% sodium citrate) for 2 min on ice, and then incubated with TUNEL reaction mixture (*In Situ* Cell Death Detection Kit (Roche, Germany)) in a humid chamber at 37°C for 60 min. Afterwards, the sections were incubated with mouse anti-NeuN (Abcam, CA, USA) overnight at 4°C and then mounted with DAPI (Beyotime, China) for 5 min in the dark. All the double-labeled cells (NeuN/TUNEL) were carefully counted by three investigators to analyze the neuronal apoptosis in the hippocampal CA1 subarea during CCH.

### Caspase Activity

Caspase-3, -8 and -9 activity was measured by colorimetric assay kits (EMD Millipore, Billerica, MA, USA) according to the manufactures’ protocol. In brief, the hippocampal CA1 tissue was carefully isolated from the rat brain (sham group: *n* = 6; 2VO group: *n* = 6 per time point) under microscope and homogenized with 1 ml ice-cold lysis buffer supplied with the kit. After centrifugation (10,000 rpm for 15 min) and assessment of protein concentration, the samples were assayed for caspase activity based on the detection of the chromophore p-nitroaniline (pNA) after cleavage from the labeled substrates (Ercan et al., 2015). The cleavage of synthetic caspase-3, -8 and -9 were spectrophotometrically detected at 405 nm in a microplate reader. The value for the sham group was defined as 1 and the ratio of optical density (OD) was finally used in the present study to show the caspase activity at different time points during CCH.

### Statistical Analysis

SPSS 19.0.0 was used for statistical analysis of the data. Data were presented as the mean ± SEM. Repeated measures were used to analyze time latency and swimming speed in MWM tests, and comparisons in the individual day were analyzed by Student’s *t*-test, which was also used to analyze results from time spent in the target quadrant and the number of target crossings. Other multiple comparisons were analyzed using one-way ANOVA followed by *post hoc* Bonferroni’s test. Values of *P* < 0.05 were considered to be statistically significant.

## Results

### Cognitive Deficits and Neuronal Loss in 2VO Rats

**Figure 1:**
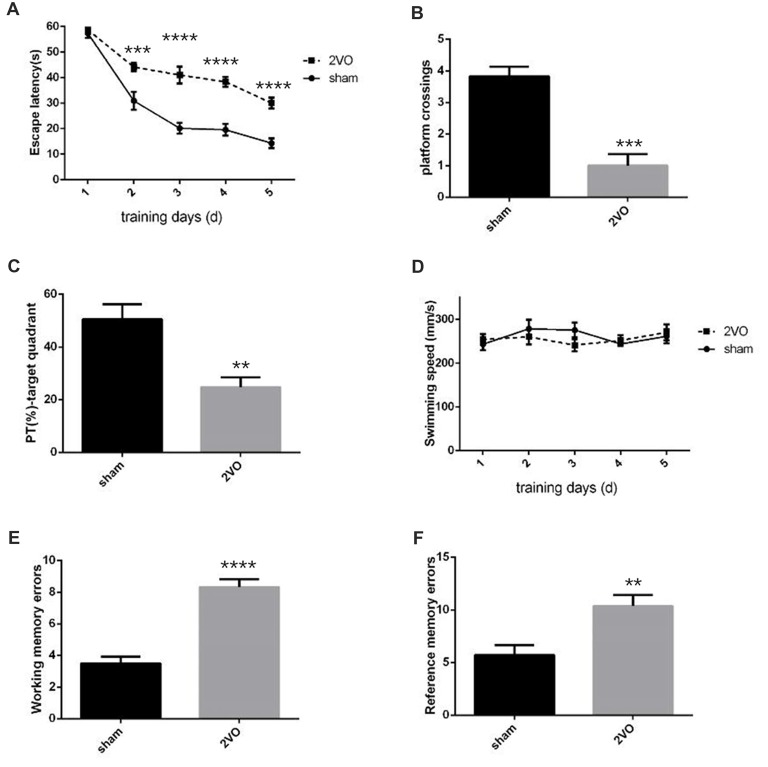
Decreased spatial learning and memory abilities in permanent bilateral common carotid occlusion (2VO) rats. **(A)** The escape latency in the hidden platform task. **(B)** The number of platform crossings in the single probe task. **(C)** Percentage of time spent in the target quadrant during the single probe task. **(D)** The swimming speed. **(E)** The cumulative data of working memory errors in Radial arm water maze (RAWM). **(F)** The cumulative data of reference memory errors in RAWM. Working memory errors defined as re-entries into arms which had previously been explored. Reference memory errors scored as first entries into arms not containing the platform. Data are expressed as mean ± SEM. *n* = 6 for each group. The changes in escape latency and swimming speed were first evaluated using repeated measures, then comparisons between the two groups were analyzed using Student’s *t*-test, which is also used for others. **p < 0.01, ***p < 0.001, ****p < 0.0001 vs. sham group.

**Figure 2:**
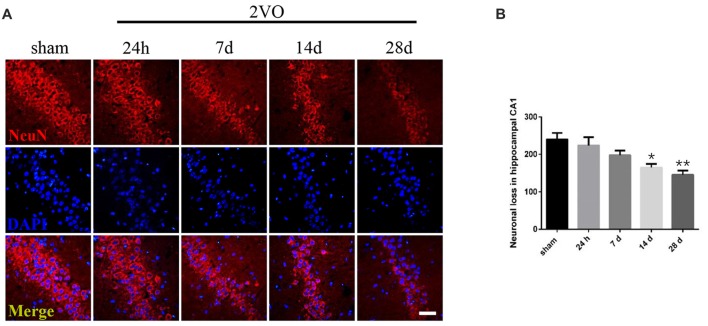
Neuronal loss in the hippocampal CA1 subarea at different time points during Chronic Cerebral Hypoperfusion (CCH). **(A)** Neuronal nuclei (NeuN) immunostaining in the sham group and in the 2VO group (24 h, 7 days, 14 days, 28 days). Horizontal bar = 50 μm. **(B)** The quantification of NeuN-positive cells. Data are expressed as mean ± SEM. *n* = 6 for each group. Multiple comparisons were evaluated using one-way ANOVA. Individual comparisons were analyzed by *post hoc* Bonferroni’s test. *p < 0.05, **p < 0.01 vs. sham group.

Behavioral assessments were performed on day 28 after surgery. For MWM tests (Figures 1A–D), the 2VO group showed obvious longer time latencies in comparison to the sham group (training day 2: *P* = 0.0001 < 0.001; training day 3: *P* < 0.0001; training day 4: *P* < 0.0001; training day 5: *P* < 0.0001). Besides, the percentage of time (PT (%)) and the number of platform crossings in the 2VO group increased significantly compared to the sham group (PT (%): *P* = 0.0035 < 0.01; platform crossings: *P* = 0.0001 < 0.001). No significant difference was found in the swimming speed between the two groups. For RAWM tests (Figures 1E,F), 2VO group showed remarkably increased numbers of working memory errors (*P* < 0.0001) and reference memory errors (*P* = 0.0082 < 0.01) compared to the sham group. In addition, we further performed NeuN immunostaining to see the neuronal loss in the hippocampal CA1 subarea at different time points during CCH (Figures 2A,B). Although no significant difference was found between the sham group and 2VO group at 7 days (*P* > 0.05), reduced NeuN-positive cells were clearly observed at 14 days (*P* = 0.0338 < 0.05) and 28 days (*P* = 0.0026 < 0.01).

### The Expression of Hippocampal CA1 NRG1/ErbB4 During CCH

**Figure 3:**
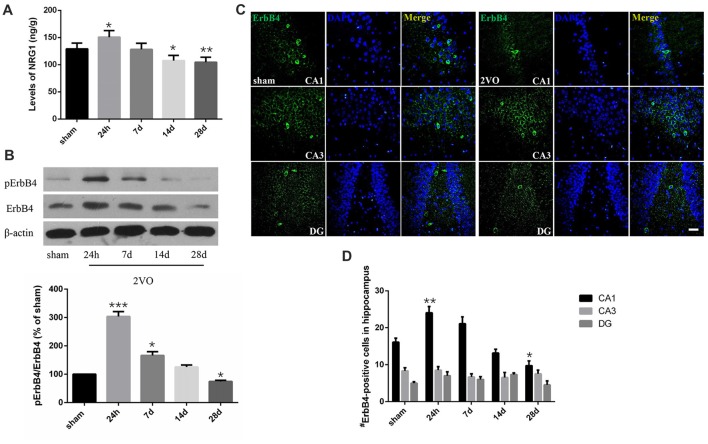
Dynamic changes of NRG1 and pErbB4/ErbB4 in hippocampal CA1 subarea at different time points during CCH. **(A)** The concentration of NRG1 detected by Enzyme-Linked Immunosorbent Assay (ELISA). **(B)** Relative protein expression and the quantification of pErbB4/ErbB4. Densities were normalized to β-actin and the value for the sham group was defined as 100%. **(C)** Representative images of ErbB4-immunostaining in the hippocampal subareas. Horizontal bar = 50 μm. **(D)** Quantification of ErbB4-positive cells. The data are expressed as mean ± SEM. For ELISA, western blotting and immunostaining, *n* = 6 for each group. Multiple comparisons were evaluated using one-way ANOVA. Individual comparisons were analyzed by *post hoc* Bonferroni’s test. *p < 0.05, **p < 0.01, ***p < 0.001 vs. sham group.

In order to determine the spatial and temporal changes of hippocampal NRG1/ErbB4 during CCH, ELISA, western blotting and immunofluorescence were conducted at different time points (Figure 3). ELISA results showed that the concentration of NRG1 in the 2VO group significantly improved at 24 h in 2VO rats in comparison to the sham group (*P* = 0.0173 < 0.05), and then gradually decreased from day 7 to day 28 (*P* = 0.0141 < 0.05 for 14 days; *P* = 0.0043 < 0.01 for 28 days). For western blotting, similarly, the relative protein levels of pErbB4/ErbB4 in the 2VO group increased at 24 h (*P* = 0.0002 < 0.001) and 7 days (*P* = 0.0381 < 0.05) compared to that of the sham group, and gradually reduced later (*P* = 0.0130 < 0.05 at 28 days). Meanwhile, the population of ErbB4-positive cells in the hippocampal CA1 subarea in the 2VO group remarkably increased at 24 h compared to the sham group (*P* = 0.0056 < 0.01), and then significantly reduced on day 28 (*P* = 0.0393 < 0.05) in comparison to the sham group. However, no significant difference was found in regard to CA3 and DG.

### The Distribution of ErbB4 in Hippocampal CA1 Subarea in 2VO Rats

**Figure 4:**
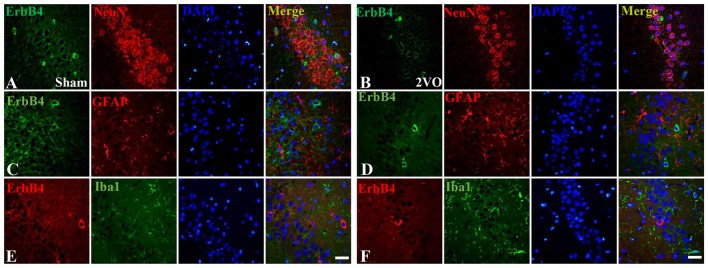
The co-localization of ErbB4 with neurons, microglia and astrocytes at 28 days. The representative images in the sham group are listed in the left four columns **(A,C,E)** and the images in the 2VO group are listed in the right **(B,D,F)**. Horizontal bar = 50 μm. Note that ErbB4 is mainly expressed in NeuN-positive cells.

**Figure 5:**
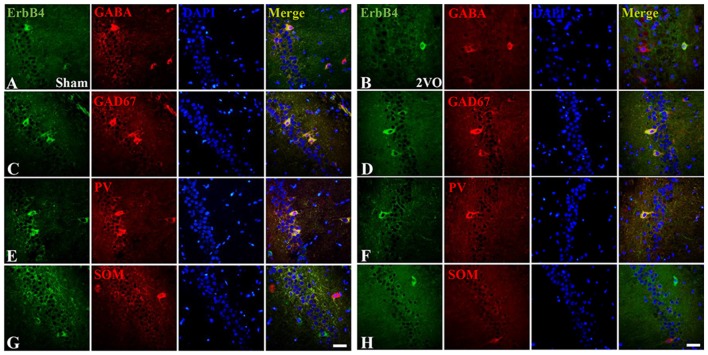
The co-localization of ErbB4 with GABAergic cells (GABA, GAD67, Parvalbumin (PV) and Somatostatin (SOM)) at 28 days. The representative images in the sham group are listed in the left four columns **(A,C,E,G)** and the images in the 2VO group are listed in the right **(B,D,F,H)**. Horizontal bar = 50 μm. Note that ErbB4 is expressed in GABA, GAD67 and PV-positive cells, but not in SOM-positive cells.

**Figure 6:**
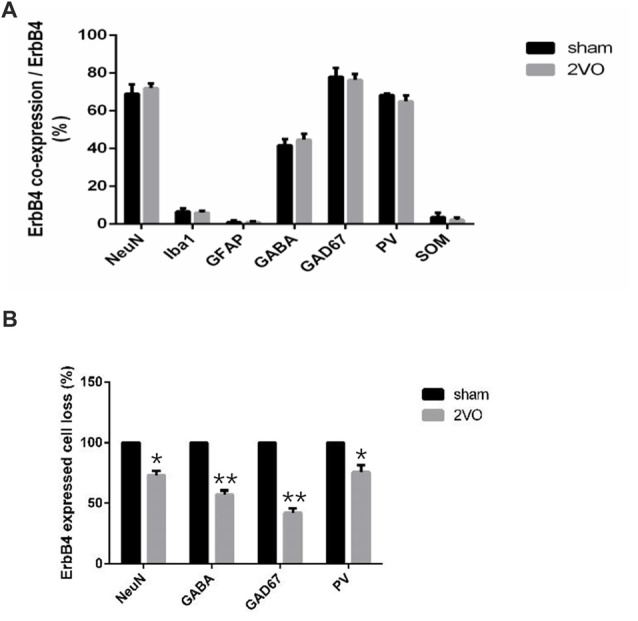
The distribution pattern of ErbB4 in the hippocampal CA1 subarea after CCH. **(A)** Comparison of the co-expression pattern of ErbB4 between the sham group and the 2VO group at 28 days. No significant difference was found between the two groups. **(B)** The quantification of ErbB4-expressed cell loss (NeuN, GABA, GAD67, PV) at 28 days. The value for the sham group was defined as 100%. Data are expressed as mean ± SEM. Comparisons in the two groups (*n* = 6 for each group) were analyzed by Student’s *t*-test. *p < 0.05, **p < 0.01 vs. sham group.

First, we investigated the co-expression of ErbB4 and the main cell-types in the brain including neurons, microglia and astrocytes on day 28 (Figure 4). Double-immunofluorescence revealed that ErbB4 was largely expressed in the NeuN-positive cells, but rarely in Iba1 and GFAP-positive cells, indicating that the ErbB4 expression was mainly located in neurons. To further determine the distribution of ErbB4 in GABAergic cell types, GABA, GAD67, PV and SOM antibodies were used here (Figure 5). As expected, ErbB4 is largely expressed in the GAD67 and PV-positive cells, but rarely expressed in SOM-positive cells in the hippocampal CA1 subarea. In addition, the difference of expression patterns between the sham group and the 2VO group was analyzed using Student’s *t*-test (Figure 6A) and no significant difference was found between the two groups, indicating that the co-expression pattern of ErbB4 remained unchanged during CCH. Furthermore, ErbB4 expressed neurons (*P* = 0.0411 < 0.05) and GABAergic cells (*P* = 0.0091 < 0.01 for GABA; *P* = 0.0071 < 0.01 for GAD67; *P* = 0.0233 < 0.05 for PV) in the 2VO group significantly reduced at 28 days compared to the sham group (Figure 6B), which may contribute to the decreased expression of hippocampal ErbB4 in 2VO rats.

### The Expression of NRG1/ErbB4 Directly Correlated With Neuronal Apoptosis, But Not With Glial Activation During CCH

**Figure 7:**
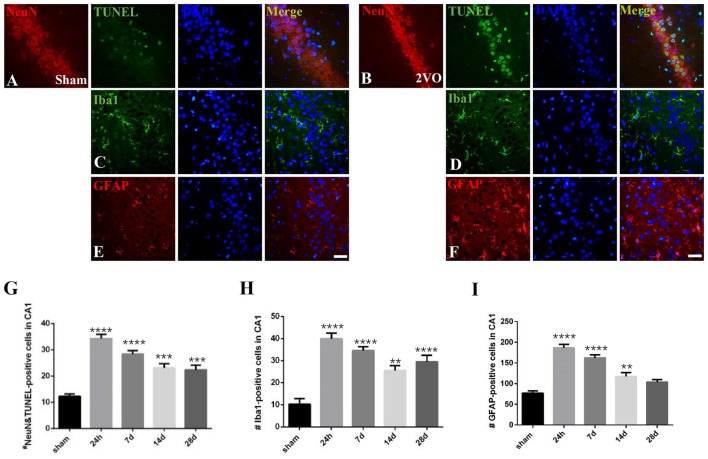
The dynamic changes of neuronal apoptosis and glial activation during CCH. **(A–F)** Representative images of ErbB4/TUNEL, NeuN/TUNEL, Iba1 and GFAP-positive cells in the sham group (left) and 2VO group (right) at 28 days. Horizontal bar = 50 μm. **(G–I)** The quantification of NeuN/TUNEL, Iba1 and GFAP-positive cells at different time points. The data are expressed as mean ± SEM. *n* = 6 for each group. Multiple comparisons were evaluated using one-way ANOVA. Individual comparisons were analyzed by *post hoc* Bonferroni’s test. **p < 0.01, ***p < 0.001, ****p < 0.0001 vs. sham group.

We analyzed the dynamic changes of NeuN/TUNEL, Iba1 and GFAP-positive cells at different time points (Figure 7). The level of NeuN/TUNEL positive cells significantly increased and peaked at 24 h (*P* < 0.0001 vs. sham group), and then remained high after that (*P* < 0.0001 for 7 days; *P* = 0.0002 < 0.001 for 14 days; *P* = 0.0006 < 0.001 for 28 days). For glial activation, similarly, the positive cells dramatically increased and peaked at 24 h in the 2VO group (Iba1: *P* < 0.0001 vs. sham group; GFAP: *P* < 0.0001 vs. sham group), and then remained at a relatively high level (Iba1: *P* < 0.0001 for 7 days and 28 days, *P* = 0.0014 < 0.01 for 14 days; GFAP: *P* < 0.0001 for 7 days, *P* = 0.0068 < 0.01 for 14 days).

Pearson’s correlation was used here to detect the possible relationship between the expression of hippocampal CA1 NRG1/ErbB4 and neuronal apoptosis and glial activation. The concentration of NRG1 and the numbers of immunostained cells in the hippocampal CA1 subarea counted at different time points (24 h, 7 days, 14 days, 28 days) were analyzed (Table 1). Interestingly, both the concentration of NRG1 and the number of ErbB4 immunostained cells were positively correlated with TUNEL-positive cells (NRG1: *r* = 0.869, *P* < 0.05; ErbB4: *r* = 0.859, *P* < 0.05), but no significant results were found in regard to Iba1 or GFAP (*P* > 0.05).

**Table 1:** The concentration of Neuregulin1 (NRG1) (detected by Enzyme-Linked Immunosorbent Assay (ELISA)) and the number of immunostained cells (hippocampal CA1 subarea) during chronic cerebral hypoperfusion (CCH).

Time point	NRG1 (ng/g)^a^	ErbB4^b^	NeuN & TUNEL^c^	Iba1^d^	GFAP^e^
Sham	129.33 ± 4.10	16.12 ± 0.79	12.33 ± 0.81	10.23 ± 1.157	77.00 ± 5.20
24 h	150.72 ± 4.98	24.06 ± 1.70	34.33 ± 1.54	39.95 ± 2.47	186.30 ± 8.86
7 days	128.31 ± 4.49	21.10 ± 1.85	28.33 ± 1.33	34.57 ± 1.79	162.31 ± 6.98
14 days	107.53 ± 3.97	13.12 ± 1.01	23.17 ± 1.54	25.50 ± 2.19	117.49 ± 9.52
28 days	104.71 ± 3.72	9.74 ± 1.29	22.33 ± 1.86	29.47 ± 2.90	103.69 ± 6.15

*Data are expressed as mean ± SEM. n = 6 for each group. Pearson’s correlational analysis showed significant results between ^a^ and ^c^ (r = 0.869, P < 0.05), ^b^ and ^c^ (r = 0.859, P < 0.05). But no significant correlation was found between ^a^ and ^d^ (r = 0.020, P > 0.05), ^b^ and ^d^ (r = 0.078, P > 0.05), ^a^ and ^e^ (P > 0.05), ^b^ and ^e^ (P > 0.05)*.

**Figure 8:**
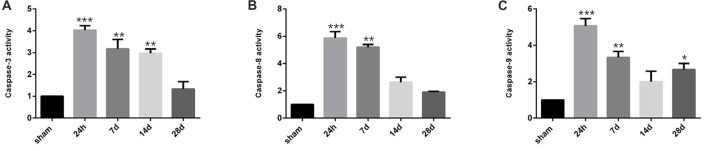
Caspase activity in the hippocampal CA1 subarea at different time points during CCH. **(A)** The quantification of caspase-3 activity. **(B)** The quantification of caspase-8 activity. **(C)** The quantification of caspase-9 activity. Data are expressed as mean ± SEM. *n* = 6 for each group. The values in the sham group was defined as 100%. Multiple comparisons were evaluated using one-way ANOVA. Individual comparisons were analyzed by *post hoc* Bonferroni’s test. *p < 0.05, **p < 0.01, ***p < 0.001 vs. sham group.

Since positive correlation exists between the NRG1/ErbB4 expression and TUNEL/NeuN positive cell counts, we further performed caspase activity assay to validate the role of NRG1/ErbB4 in hippocampal apoptosis (Figure 8). And we found the caspase-3, -8 and -9 activities peaked at 24 h post-operation (*P* < 0.001) compared to the sham group and gradually decreased after that. The temporal profile of caspase activation seemed to coincide with the expression of NRG1/ErbB4 and Pearson’s correlation further revealed that caspase-3 activity positively correlated with the levels of NRG1 (*r* = 0.671, *P* < 0.05).

## Discussion

Numerous studies have demonstrated the neuroprotective and anti-inflammatory effects of NRG1, and even reported improved cognitive function after NRG1 administration (Guan et al., 2015; Lu et al., 2016). And in the present study, we explored the expression pattern of hippocampal NRG1/ErbB4 during CCH and its possible relationship with neuronal apoptosis and glial activation. We found that: (1) the expression of NRG1 and pErbB4/ErbB4 peaked in the acute phase and then decreased in the chronic phase of CCH; (2) ErbB4 expressed neurons and selective types of GABAergic cells decreased after CCH, but the distribution pattern of ErbB4 remained unchanged; and (3) the expression of hippocampal NRG1/ErbB4 positively correlated with the degree of neuronal apoptosis (both NeuN/TUNEL immunostaining and caspase-3 activity), but not with glial activation according to Pearson’s correlation.

To begin with, thorough analysis of the expression pattern of NRG1/ErbB4 in the hippocampal CA1 subarea is crucial for us to explore their possible involvement in the pathogenesis of CCH. According to the previous studies, the expression of NRG1 and ErbB4 receptors significantly increased in the acute phase in cerebral ischemia (24 h after surgery) and in traumatic brain injury (Erlich et al., 2000; Tokita et al., 2001). Meanwhile, a number of studies have also reported down-regulated expression of NRG1/ErbB4 signaling in sepsis-associated encephalopathy (SAE) 20 days after model establishment (Gao et al., 2017). In agreement of these studies, here we found that hippocampal CA1 NRG1 and pErbB4/ErbB4 expression changed in a time-dependent manner during CCH. Lu et al. (2016) recently reported the decreased expression of membrane ErbB4 receptors in neurons in cerebral ischemia, suggesting that recruitment or translocation of ErbB4 stimulated by NRG1 treatment is disrupted (Ma et al., 2003). Therefore, the specific location of ErbB4 should be noted too.

It is known that EGF and NRG1’s actions on GABAergic neurons are inconsistent among the ErbB ligands (Namba et al., 2006; Nagano et al., 2007). More specifically, exogenous EGF administration can reduce the expression of GAD67 and PV in neocortical cultures (Nawa et al., 2014), while NRG1/ErbB4 signaling seems to improve the development of GABAergic cells. We further analyzed the hippocampal expression of EGF and ErbB1 using ELISA and western blotting respectively (see details in the Supplementary Figure S1) and observed the up-regulation of EGF and ErbB1 expression during CCH, which is consistent with the previous research (Ashok et al., 2016). In view of the opposite changes of NRG1/ErbB4 and EGF/ErbB1, the expression of hippocampal NRG1/ErbB4 seems to be more directly linked with cognitive deficits in CCH. By the way, since the protein levels of NRG1 and ErbB4 in the hippocampus changed after 2VO surgery in our study, the transcription/translation level of them could probably present corresponding changes as well. As far as we know, the mRNA levels of NRG1 and ErbB4 have not been investigated during CCH, but similar changes have been reported in other animal models of neurodegenerative diseases (Croll et al., 1998; Dickerson et al., 2009). Interestingly, Dickerson et al. (2009) reported that the ErbB4 mRNA transcripts in rat brain selectively decreased prior to other biochemical changes in aged rats, indicating that the change of ErbB4 in translation level, rather than the protein level, can predict functional changes in neurodegenerative disorders. Therefore, time-dependent changes of NRG1/ErbB4 mRNA levels should be systematically investigated in the future.

Emerging evidence has showed the protective role of NRG1/ErbB4 in neuronal and non-neuronal cells in the hippocampal subareas (Shyu et al., 2004; Simmons et al., 2016). To further determine the role of NRG1/ErbB4 signaling on different cell types, we performed double-immunofluorescence to investigate the distribution of ErbB4 receptors and found that ErbB4 largely co-expressed in neurons (NeuN-positive cells), especially selective subtypes of GABAergic interneurons (GAD67 and PV-positive cells, but not in SOM-positive cells) in the hippocampal CA1 region. These findings were consistent with others (Neddens and Buonanno, 2010; Vullhorst et al., 2009; Li et al., 2014). Besides, we found the distribution of ErbB4 remained unchanged during CCH, while the ErbB4 expressed neurons and selective types of GABAergic cells significantly decreased in the chronic phase of CCH. In fact, we have counted the GABAergic cells in hippocampal CA1 subarea in CCH before and found decreased expression of GABA, GAD67 and GABA_B_Rs in hippocampal CA1 region (Long et al., 2015). It is likely that the remarkable decrease of ErbB4 expression in the chronic phase is caused by the reduction of these neurons and GABAergic cells (especially GAD67 and PV).

The long-lasting cognitive impairments, as well as improved degrees of hippocampal CA1 neuronal apoptosis and neuroinflammation, in CCH have been well-established over the years (Cechetti et al., 2012; Du et al., 2017), but there are still some details that should be addressed in this study. First, Ji et al. (2010) reported that the expression of hippocampal NRG1/ErbB4 significantly reduced during spatial learning tests (Tian et al., 2016). Therefore, rats that underwent cognitive tests (WMW and RAWM) in this study were not used for biochemical analysis. Second, increased neuronal death and glial activation in CCH have been reported to present early at 7 days post-operation and continue for long (Cechetti et al., 2012). However, some of the molecular changes in the hippocampus seem to change in a different way. For example, the expression of anti-apoptotic factors (such as Bcl-2) was reported to increase only in the acute phase, followed by a remarkable reduction in the chronic phase of CCH (Bang et al., 2013; Yang et al., 2013). We also found transiently increased expression of NRG1/ErbB4 signaling in the current study. The mismatch of these factors in the chronic phase of CCH may further contribute to cognitive impairment and neuropathologic changes during CCH.

Although the mismatch occurs between the NRG1/ ErbB4 expression and neuropathologic changes in the chronic phase of CCH, all these factors seemed to increase in the acute phase, and then gradually decreased later during CCH. Pearson’s correlation in our study further revealed that both the expression of NRG1 and ErbB4 positively correlated with NeuN/TUNEL cell counts during CCH. Besides, delayed hippocampal neuronal death after CCH is mediated, at least in part, through the activation of terminal caspases, particularly caspase-3 (Ji et al., 2010). Caspase-8 and caspase-9 probably mediate the activation of caspase-3 under an ischemic condition via extrinsic and intrinsic pathways respectively (Cao et al., 2002). Consistently, we found up-regulated expression of caspase activities and positive correlation between caspase-3 activity and NRG1 expression. Taken together, our results suggest the protective role of NRG1/ErbB4 signaling in CCH is closely related to direct inhibition of neuronal apoptosis, thus providing a friendly environment for cognitive improvement.

By the way, some studies have demonstrated that NRG1/ErbB signaling could improve proliferation in microglia and astrocytes (Calvo et al., 2011; Simmons et al., 2016), while both we and others found that only a few ErbB4 receptors co-expressed in glial cells, especially GFAP-positive astrocytes (Xu and Ford, 2005). Furthermore, Ghashghaei et al. (2006) further reported that these cells do not respond to exogenous NRG1 infusion in the brain. These findings may explain the reason the NRG1/ErbB4 signaling failed to correlate with glial activation. Finally, in order to further determine the neuroprotective effects of NRG1/ErbB4 signaling during CCH, the bilateral regulation of NRG1/ErbB4 signaling using an agonist/antagonist and ErbB4 knockout rats can be more useful.

## Author Contributions

YH and RC, performed the experiments, analyzed the data and drafted the manuscript. QL and WL, designed the experiments and approved the final manuscript. LW and XY, contributed reagents/materials/analysis tools. All authors read and approved the final manuscript.

## Conflict of Interest Statement

The authors declare that the research was conducted in the absence of any commercial or financial relationships that could be construed as a potential conflict of interest.

## Supplementary Material

FIGURE S1 Analysis of the hippocampal expression of epidermal growth factor (EGF) and ErbB1 using Enzyme-Linked Immunosorbent Assay (ELISA) and western blotting.
