# Supplementary figures and images for: The Expression of Hippocampal NRG1/ErbB4 Correlates With Neuronal Apoptosis, but Not With Glial Activation During Chronic Cerebral Hypoperfusion
Source: Front Aging Neurosci. 2018 May 23;10:149. doi: 10.3389/fnagi.2018.00149 (PMC5974051; doi:10.3389/fnagi.2018.00149)

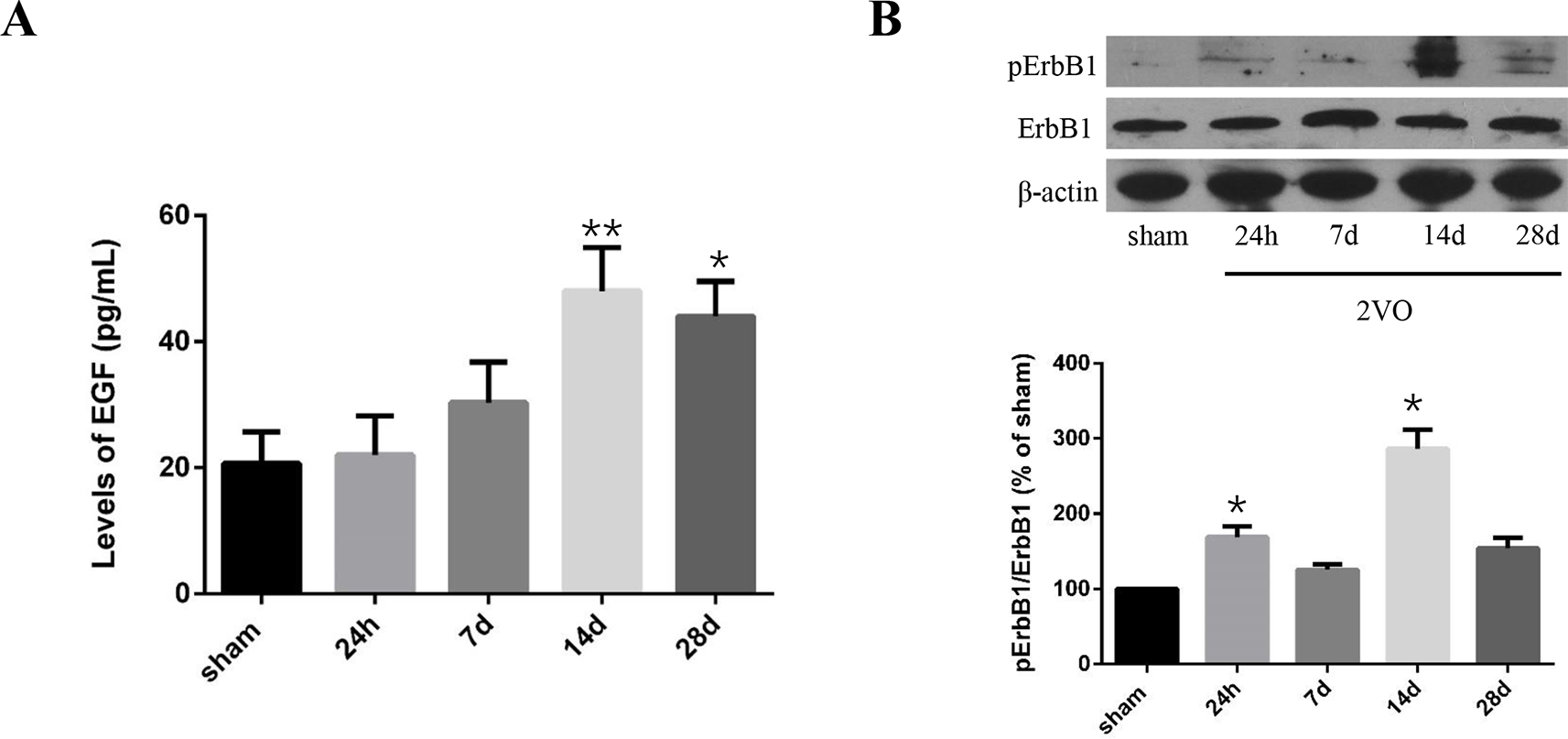

Supplement: FIGURE S1 — Analysis of the hippocampal expression of epidermal growth factor (EGF) and ErbB1 using Enzyme-Linked Immunosorbent Assay (ELISA) and western blotting. [file Image_1.TIF]
